# Longitudinal Changes in Pitch-Related Acoustic Characteristics of the Voice Throughout the Menstrual Cycle: Observational Study

**DOI:** 10.2196/65448

**Published:** 2025-01-09

**Authors:** Jaycee Kaufman, Jouhyun Jeon, Jessica Oreskovic, Anirudh Thommandram, Yan Fossat

**Affiliations:** 1 Klick Applied Sciences, Klick Health, Toronto, ON, Canada; 2 Faculty of Science, University of Ontario Institute of Technology, Oshawa, ON, Canada

**Keywords:** menstrual cycle, women's health, voice, acoustic analysis, longitudinal observational study, fertility tracking, fertility, reproductive health, feasibility, voice recording, vocal pitch, follicular, luteal phase, fertility status, mobile phone

## Abstract

**Background:**

Identifying subtle changes in the menstrual cycle is crucial for effective fertility tracking and understanding reproductive health.

**Objective:**

The aim of the study is to explore how fundamental frequency features vary between menstrual phases using daily voice recordings.

**Methods:**

This study analyzed smartphone-collected voice recordings from 16 naturally cycling female participants, collected every day for 1 full menstrual cycle. Fundamental frequency features (mean, SD, 5th percentile, and 95th percentile) were extracted from each voice recording. Ovulation was estimated using luteinizing hormone urine tests taken every morning. The analysis included comparisons of these features between the follicular and luteal phases and the application of changepoint detection algorithms to assess changes and pinpoint the day in which the shifts in vocal pitch occur.

**Results:**

The fundamental frequency SD was 9.0% (SD 2.9%) lower in the luteal phase compared to the follicular phase (95% CI 3.4%‐14.7%; *P*=.002), and the 5th percentile of the fundamental frequency was 8.8% (SD 3.6%) higher (95% CI 1.7%‐16.0%; *P*=.01). No significant differences were found between phases in mean fundamental frequency or the 95th percentile of the fundamental frequency (*P*=.65 and *P*=.07). Changepoint detection, applied separately to each feature, identified the point in time when vocal frequency behaviors shifted. For the fundamental frequency SD and 5th percentile, 81% (n=13) of participants exhibited shifts within the fertile window (*P*=.03). In comparison, only 63% (n=10; *P*=.24) and 50% (n=8; *P*=.50) of participants had shifts in the fertile window for the mean and 95th percentile of the fundamental frequency, respectively.

**Conclusions:**

These findings indicate that subtle variations in vocal pitch may reflect changes associated with the menstrual cycle, suggesting the potential for developing a noninvasive and convenient method for monitoring reproductive health. Changepoint detection may provide a promising avenue for future work in longitudinal fertility analysis.

## Introduction

The menstrual cycle is a recurring sequence of physiological changes regulated by hormonal fluctuations that prepare the body for potential pregnancy. This cycle is crucial for women’s health, as it influences reproductive function, affects various aspects of physical and emotional well-being, and serves as an indicator of overall health. Irregularities or disruptions in the menstrual cycle can signal underlying health issues, making its regular monitoring and understanding essential for women’s health care. The female menstrual cycle is broken into 2 distinct phases: the follicular phase, in which hormonal estrogen levels rise, and the luteal phase, in which estrogen levels begin to decrease, and progesterone is elevated. An important step in the evaluation and management of female fertility and pregnancy is awareness of the timing of ovulation, the release of an egg from the ovary for conception. Ovulation occurs between the follicular and luteal phases and is initiated by high estrogen and luteinizing hormone (LH). The fertile window, or the time during the menstrual cycle when conception is most likely, encompasses the days just before and during ovulation. Follicle tracking through ultrasound is a common fertility procedure in a clinical setting and is considered an accurate method to evaluate the true ovulation timing. However, it comes with significant patient burden and cost. Currently, ovulation detection can be estimated at home through tracking the length of time between periods (calendar method) and assessing the day-to-day changes of cervical mucus, basal body temperature (BBT), and estrogen, progesterone and LH measurements [1].

The current at-home ovulation detection methods come with their own drawbacks. Hormone measurements and BBT recordings require the purchase of additional devices and materials. Cervical mucus monitoring requires proper education on the different appearances and consistencies throughout the menstrual cycle. Perhaps, the easiest and most accessible method, the calendar method, is widely available through various menstrual tracking apps [2]. Both the predicted day of ovulation and the estimated fertile window can be provided; however, the variation in ovulatory days is distinct. It was shown that the most common predicted fertile window days only included the day of ovulation itself for 65% of female participants [2], yielding some uncertainty in the reliability of this method.

An interesting supplement to the calendar method may be achieved via the assessment of voice throughout the menstrual cycle. Increased estrogen, such as what is seen immediately preceding ovulation, leads to increased mucus secretion by the granular glands in the respiratory tract and increased cell permeability. Conversely, laryngeal tissues begin to absorb water, as estrogen levels decrease in the luteal phase, ultimately causing mucosal edema, vascular congestion, and increased vocal fold mass [3,4]. These changes have been associated with decreased vocal range, vocal and pitch instability, vocal fatigue, and reduced power and efficiency in the voice [5,6]. As such, voice-based fertility analysis may capture physiological changes throughout the menstrual cycle, with the added benefit of convenient smartphone-based data collection, similar to the accessibility offered by the calendar method.

While assessing prior work, it is important to note the variation in methodology and subsequent results. An early study evaluated speech recordings of the vowel sounds /a/, /i/, and /u/ from 20 undergraduate female participants. Voice was recorded twice throughout the menstrual—once at ovulation and once 2‐3 days prior to the onset of menses. There was no difference in hoarseness or fundamental frequency (F0) between the 2 recordings, a result that was confirmed with a validation sample of 27 additional undergraduate female participants [7]. Similar methodology (recording once during menstruation, immediately following menstruation, midcycle, and premenstruation) with vowel sounds [8,9], counting from 1 to 10 [10], and free speech [11] also yielded no significant changes in F0. Conversely, a study conducted with 30 participants recording the vowel sound /a/ once a week for 4 weeks reported a significant increase in F0 in the ovulatory period and a decrease in F0 in the luteal period compared to the other recording time points [12], and high-fertility recordings of a spoken introductory sentence from 69 participants were significantly higher in F0 than in recordings obtained in a low-fertility period [8]. Looking at other acoustic parameters, counting from 1 to 10 yielded significant differences in shimmer (sound wave amplitude variation) and frequency variation between periods of high fertility and low fertility [10,13], and the vowel sounds /a/, /e/, /i/, /o/, and /u/ collected once each within the fertile window, luteal phase, and during menstruation from 44 participants indicated an increased minimum F0 in the fertile window [14]. In addition, a study conducted on 23 female participants recording 1 minute of free speech every day for 1 menstrual cycle showed marginally significant F0 variation with an increase prior to and a distinct drop during ovulation [15]. Furthermore, a study conducted on 17 naturally cycling female participants reported that female voices were rated more attractive in periods of high fertility when tasked with a counting task at 4 equally spaced time points throughout their menstrual cycle [16].

One of the drawbacks of these studies is that, apart from a single study [15], voice recordings were collected at a single time point within a menstrual phase; thus, day-to-day changes within the voice were notably not assessed. Ovulation itself is a result of longitudinal changes in hormone values compared to the previous days. Therefore, it is essential to trace day-to-day changes of acoustic characteristics to understand the effect of ovulation on voice signals.

In this study, we examined longitudinal changes in pitch-related acoustic characteristics using daily smartphone recordings of spoken sentences from 16 healthy female participants. Our overall goal was to determine how these vocal features change in relation to the LH surge and ovulation. We hypothesize that there are temporal changes in the F0 features associated with the menstrual cycle. The analysis is thus split into two sections:

1. Replication of the day-to-day voice change analysis conducted by Fischer et al [15] using smartphone-recorded (real-world) voice segments. The feature set will be expanded from F0 and F0 variation to include metrics denoting the minimum and maximum F0 values within a recording.
2. Employment of changepoint detection, which will be used to understand the statistical moment in which the participant voices change from one behavior to another. We hypothesize that for features significantly different between the follicular and luteal phases, this day will align with the switch between menstrual phases (ovulation). It may also provide insight into how voice may be used as a fertility indicator in future work.

## Methods

### Study Design and Participants

In total, 19 English-speaking, cis-gender female participants were recruited from Klick Health, a technology, media, and research company in the health care sector based in Toronto, Canada. Recruitment information was posted on the company’s internal communication platform, and interested parties contacted our research team privately for more information and to sign the informed consent. After the informed consent was signed, participants were onboarded through either a video call or in person. As part of the onboarding, participants were provided instructions on how to conduct the urine tests, take their BBT, and record their voice through the research app.

All participants reported a natural menstrual cycle without taking hormonal birth control or medication that would affect the menstrual cycle and reported their menstrual cycle as consistent (1‐2 days variation per cycle) or somewhat consistent (3‐4 days variation per cycle). Participants were instructed to take their BBT, self-administer a consumer-grade LH urine test (Easy@Home Ovulation Tests; Easy@Home Fertility), and record themselves saying “Hello, how are you?” every morning immediately upon waking for 1 full menstrual cycle. The decision to use a fixed phrase was made based on the success of previous studies in using fixed sentences and conversational speech to identify significant features between menstrual phases compared to vowel sounds [3,8,15]. Participants were instructed to record their voices in a quiet environment with no other voices present in the recording and took pictures of the LH test strips each day to record hormone test results.

The data collection started the day after the end of menstruation, and recordings were collected for 1 full menstrual cycle, ending on the last day of menstruation. Participants did not perform the hormone test on days they were menstruating but did record their voice and BBT. Data (LH test results, BBT, and voice recordings) were collected using a custom mobile app developed by Klick Applied Science that participants installed on their personal cellphones following the procedure below:

1. Participants entered their participant ID (a deidentified alphanumeric code) upon their first login to the mobile app.
2. When the app was opened, the participant selected “Record a New Entry” to input the study data for the day.
3. They then recorded the results of their BBT (to 2 decimal places), their LH ovulation test results, and their voice while holding their phone.
4. After all fields were completed, the participants could submit the data recording. Submitted data were uploaded to a secure Google Firestore database, where it could be accessed by our researchers via a private application programming interface key.

Participants were excluded if the LH peak could not be detected, either through multiple consecutive missed data recordings (n=2) or if no discernable LH rise was detected through hormone tests (n=1). In total, 16 participants were included with a total of 320 voice recordings.

### Ethical Considerations

Ethics was obtained from the Canadian SHIELD Ethics Review Board (REB tracking 2022-02-004). Informed consent was collected for each participant, and all procedures corresponded to local guidelines. Participant data were deidentified during collection. Participants were compensated US $77 for participating in the study and received an additional US $38.50 if they recorded all the voice recordings.

### Longitudinal Landscape of Voice Signals

Individual participants recorded their voices once a day, every day for 1 menstrual cycle. The average menstrual cycle length was 28.81 (SD 2.90) days. The average length of the follicular phase (including menstruation and the day of the LH surge and the day after) was 15.92 (SD 3.30) days, and the average length of the luteal phase was 12.75 (SD 1.44) days. On average, menstruation was 5.54 (SD 1.66) days and ended 9.44 (SD 3.12) days prior to the LH surge. In total, 320 voice recordings were collected. Study participants had an average age of 30.6 (SD 4.5) years and an average BMI of 22.7 (SD 2.8) kg/m^2^ and included various ethnic backgrounds (East or Southeast Asian: n=4, 25%; Jewish: n=1, 6%; Middle Eastern: n=2, 13%; South Asian: n=6, 37%; White: n=3, 19%; self-identification).

### Voice Feature Extraction and Analysis

The LH surge was defined to be when the hormone strip test line was at its maximum darkness out of all the days in the menstrual cycle and approximately equal to or greater than the darkness of the control line. The ovulation window was defined as the day of the LH peak and the day after (to cover the 24‐36 hour likelihood for ovulation). Days prior to this were classified as “follicular,” corresponding to the follicular phase in the menstrual cycle, and days following ovulation classification and occurring prior to menstruation were classified as “luteal.” Recordings collected while participants were menstruating were labeled “menstrual.” BBT was used to verify the likelihood that ovulation occurred through a sustained 0.3 °C increase in the days following the suspected ovulation day.

Each audio recording was assessed by researchers to ensure adequate recording quality and the absence of background noise. In total, 4 vocal features associated with the recording F0 were extracted from each voice recording: the mean (F0AV), SD (F0SD), 5th percentile (F0MIN), and 95th percentile (F0MAX). The 5th and 95th percentiles were chosen as robust analogs to the minimum and maximum F0 values. Voice features were extracted using librosa [17], a Python library widely used for music and audio analysis, providing tools for audio feature extraction and processing tasks. F0 values for the recording were extracted using the *pyin* function, with the specified frequency minimum of C2 (~65 Hz) and maximum of C7 (~2093 Hz). These values were chosen to encompass a wide range of vocal frequencies and are the recommended minimum and maximum frequencies for analysis [17].

Each voice feature was scaled independently for each participant. Each voice feature was minimum-maximum scaled according to the following formula:


(1) $$X_{sc}=\frac{X-X_{min}}{X_{max}-X_{min}}$$

**Figure 1.**
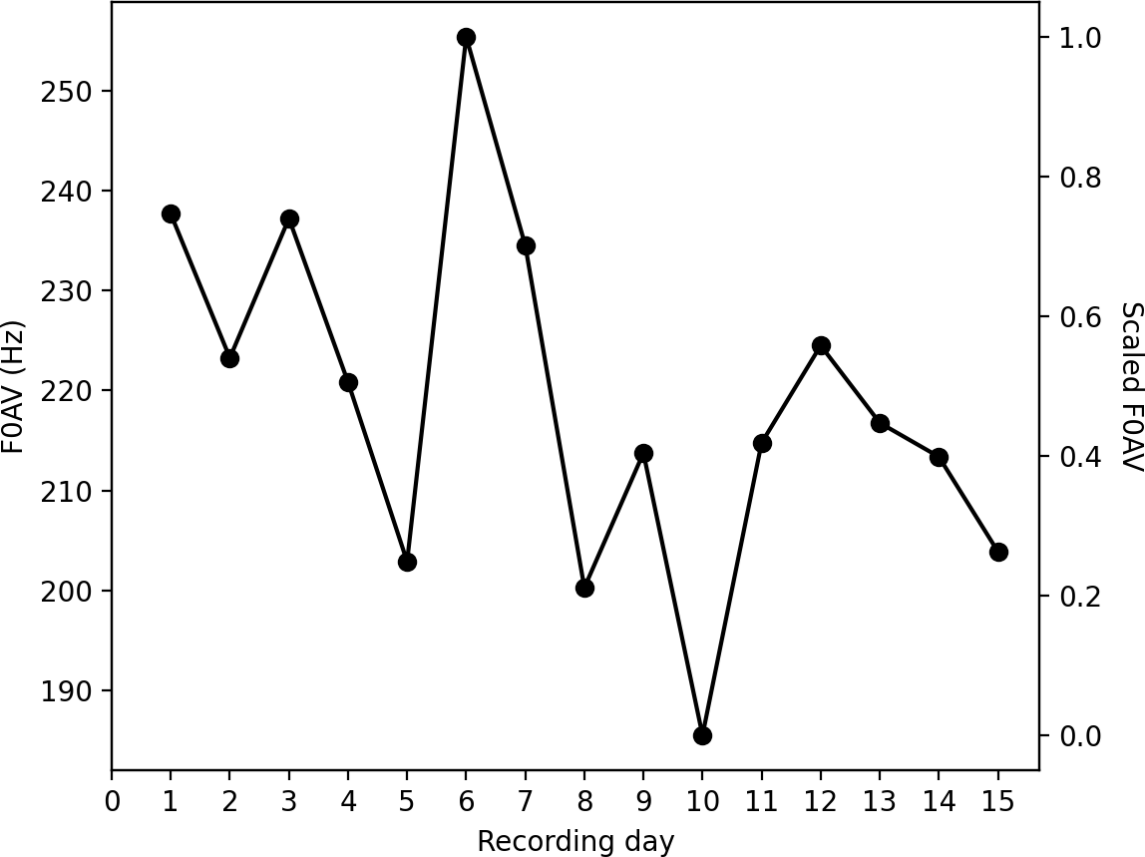
Generated mean fundamental frequency (F0AV) data to demonstrate the feature scaling process. Data are scaled for each feature within each participant, such that the daily values of a participant’s feature fall between 0 and 1.

Where *X_sc_* is the scaled feature value, *X* is the original feature value, *X_min_* is the minimum daily value of the feature throughout the participant’s menstrual cycle, and *X_max_* is the maximum daily value of the feature throughout the participant’s menstrual cycle. This scaling was conducted such that the maximum scaled feature value throughout the recording period was 1 and the minimum value was 0 (Figure 1). This was repeated for each feature, for each participant.

### Changepoint Detection

To assess the change in voice features throughout the menstrual cycle, we used changepoint detection. Changepoint detection algorithms are tools used to identify points in time where the statistical properties of a data sequence change. These changes can signify shifts in mean, variance, distribution, or other characteristics of the data and can be used to detect shifts in patient health status or the effectiveness of treatments over time [18-23]. Changepoint detection algorithms are especially valuable for identifying transitions between distinct stages [23], making them a promising tool for research on physiological changes throughout the menstrual cycle. In this study, we used the *Ruptures* package, a Python library designed for offline changepoint detection, which considers all data points within a time series to identify underlying statistical changes in the data [24]. This unsupervised algorithm does not use prior knowledge of ovulation days and is not influenced by other participants’ data, ensuring that the detection process only identifies changes in the voice recording F0 data themselves.

We applied 3 changepoint detection algorithms: dynamic programming, binary segmentation, and bottom-up segmentation. Dynamic programming explores all possible partitions of the time series to identify the optimal segmentation, constrained to detect only a single changepoint. Binary segmentation iteratively identifies the most significant changepoint in each segment but, for this study, was configured to stop after detecting the first changepoint. Bottom-up segmentation begins with many small segments and merges the segments that are most similar until only 1 changepoint remains, meeting the defined stopping criterion.

To quantify changes, we used 3 cost functions: least absolute deviation loss function (L1), least squared deviation loss function (L2), and radial basis function. The least absolute deviation cost function measures absolute differences, making it robust to outliers. The least squared deviation cost function emphasizes larger deviations by using squared differences. The radial basis function cost function captures nonlinear changes by comparing segments with a radial basis kernel. Detailed descriptions of these algorithms and cost functions are available in the *Ruptures* documentation [24].

We were not aiming to identify changes associated with menstruation so we specified the detection of a single changepoint within a window spanning from 9 days before to 9 days after the LH surge. These time points correspond to the average number of days we observed occurring prior to the LH surge following menstruation (9.4, SD 3.1 days) and the postovulatory day that the corpus luteum begins to disintegrate and hormone levels fall [25]. The algorithm was configured with a minimum distance of 3 days between any 2 breakpoints or from the start of the time series, with a jump parameter set to 1 to ensure that each day was evaluated for potential changepoints of the vocal features between menstrual phases.

### Statistical Analysis

To analyze differences between the follicular and luteal phases, 4 generalized linear mixed models were applied for the 4 F0 features. The voice feature was the dependent variable, the menstrual phase (follicular vs luteal) was the independent variable, and participant ID was a random effect to account for intraindividual variability. To avoid data that may occur between the follicular and luteal phases and ensure the clarity of phase-specific effects, days identified as ovulatory (the day of LH surge and the following day) and menstrual days were excluded from the analysis. All other data points were used. The models were estimated using restricted maximum likelihood.

To assess the significance of our changepoint findings against a null hypothesis of random results, we conducted 1-sided 2-proportion *z* tests. We compared the observed proportion of participants with a changepoint occurring before ovulation in the fertile window (days −5 to +1 relative to LH surge) to a random expectation, corresponding to 50% (n=8) of participants with a changepoint occurring in the fertile window. This proportion is based on 7 days in the fertile window of an average of 14 days in the possible prediction window (6 days prior to the LH surge maximum, the LH surge maximum, and 7 days following the LH surge maximum). The alternate hypothesis posited that the observed proportion would be higher than the random expectation due to the hormonal changes associated with the fertile window. All statistical analysis was performed in Python (version 3.12.2; Python Software Foundation) using the *statsmodels* package (*mixedlm* and *proportions_ztest* functions) [26].

## Results

### Results 1: F0 Behavior Surrounding LH Surge

**Figure 2.**
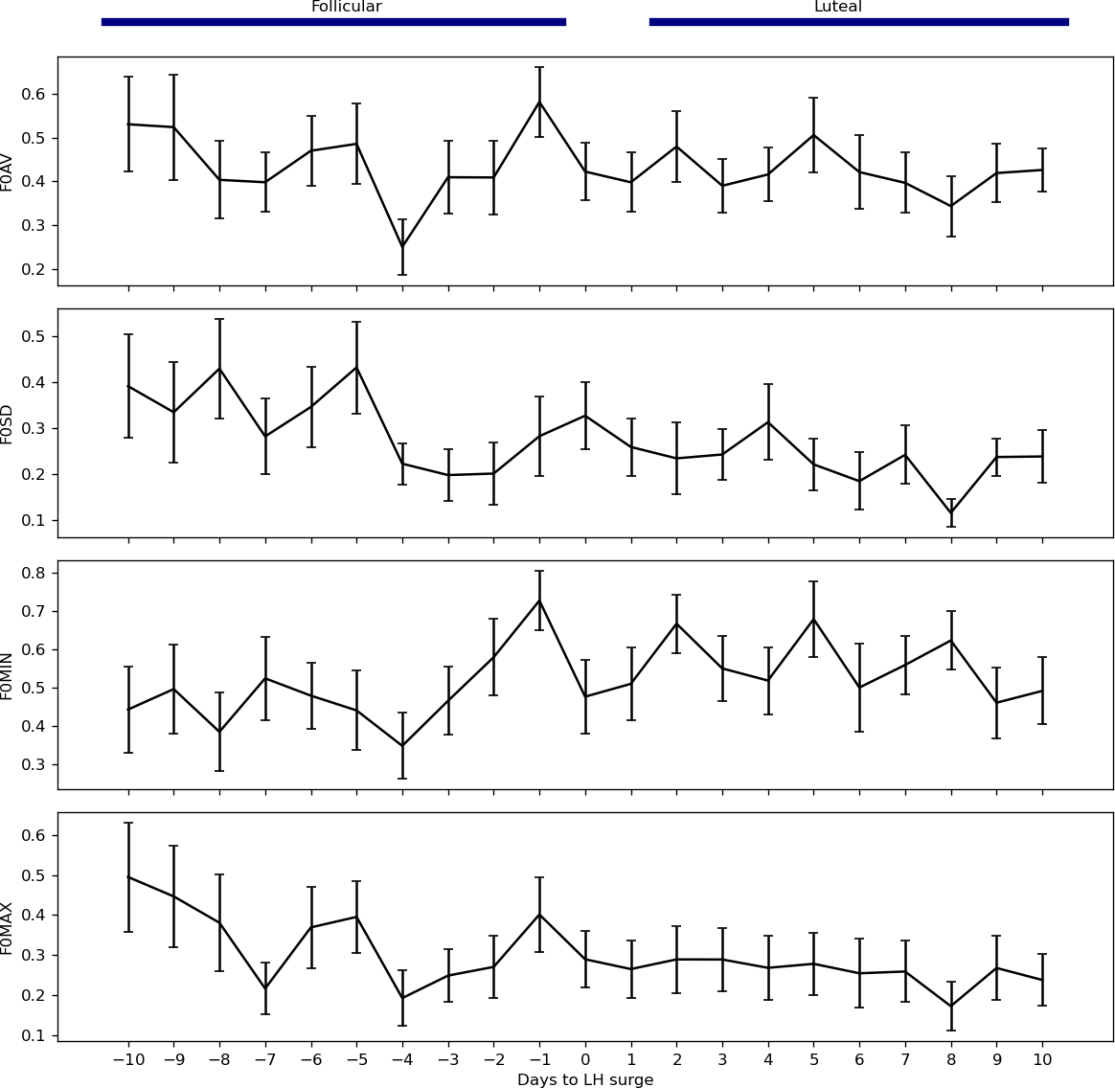
Daily patterns of fundamental frequency (**F0**) voice features—F0 average (F0AV), F0 standard deviation (F0SD), F0 5th percentile (F0MIN), and F0 95th percentile (F0MAX)—in the 9 days preceding and following the luteinizing hormone (LH) surge for all participants. Data points represent the average values of these voice features across 16 healthy female participants with regular menstrual cycles. Error bars indicate the SE of the mean.

To assess vocal dynamics consistent across participants, generalized linear mixed models were fit for each of the F0 features (F0AV, F0SD, F0MIN, and F0MAX). Overall, there was a significant difference in the F0SD and F0MIN between the follicular and luteal recording days for each participant, such that F0SD was, on average, 9.0% (SD 2.9%) lower in the luteal phase compared to the follicular phase (95% CI 3.4%‐14.7%; *P*=.002) and F0MIN was 8.8% (SD 3.6%) higher (95% CI 1.7%‐16.0%; *P*=.01). There were no significant differences between phases in F0AV or F0MAX (*P*=.65 and *P*=.07). Average daily trends of the participant-scaled F0 values are displayed in Figure 2.

### Results 2: F0 Changepoint Detection

**Figure 3.**
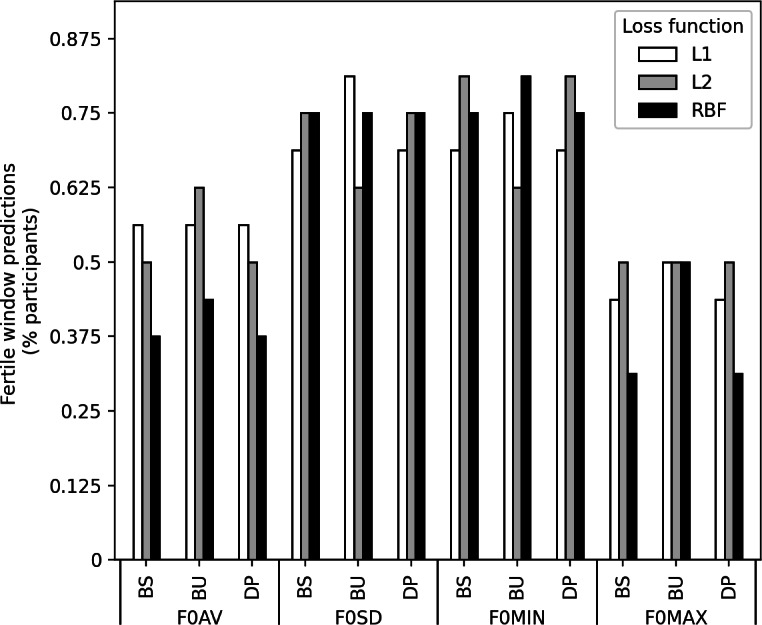
The percentage of participants with detected changepoints in the fertile window is presented for the mean, SD, 5th percentile, and 95th percentile of the fundamental frequency (F0AV, F0SD, F0MIN, and F0MAX, respectively). Changepoints were identified using 3 different detection algorithms—binary segmentation (BS), bottom-up segmentation (BU), and dynamic programming (DP)—applied to fundamental frequency features extracted from daily smartphone voice recordings of 16 naturally cycling female participants. The fertile window is defined as the 5 days leading up to and including 1 day after the luteinizing hormone surge. L1: least absolute deviation loss function; L2: least squared deviation loss function; RBF: radial basis function.

F0 feature data for each participant was fed into the changepoint detection algorithms, and the predicted changepoints are displayed in Figure 3. Menstrual phases were not added as information in the algorithms at any point, and any changepoints detected solely reflect changes in the vocal features themselves. Additionally, data from each participant were used independently for changepoint identification, ensuring that data were not shared or mixed between participants. Overall, the average (SD) detected changepoints for all algorithms were 0.20 (4.13) days following ovulation in F0AV, 1.42 (3.52) days prior to ovulation in F0SD, 0.88 (3.27) days prior to ovulation in F0MIN, and 1.31 (4.05) days following ovulation in F0MAX. As the variation in the predicted changepoints was large, we also looked where the predicted changepoints occurred specifically. We observed that up to 81% (n=13) of participants had a predicted changepoint occurring within the fertile window, or the 5 days preceding the LH surge to the 1 day following the LH surge, for both F0SD and F0MIN (*P*=.03). Meanwhile, a maximum of 63% (n=10; *P*=.24) and 50% (n=8; *P*=.50) of participants had changepoints detected in the fertile window for F0AV and F0MAX, respectively (Figure 3).

## Discussion

### Primary Findings

This study explored how vocal pitch features fluctuate across the menstrual cycle, with a focus on vocal patterns surrounding phase transitions and ovulation. Building on prior research [15], we expanded the analysis by examining a broader set of F0-related features, including minimum and maximum pitch values, using real-world voice recordings collected daily via smartphones. With more frequent recordings, we investigated whether these vocal features varied between the follicular and luteal phases, providing a more comprehensive perspective on daily vocal behavior. We further hypothesized that changepoint detection could pinpoint the transition between these phases, offering insight into the potential for voice-based markers of fertility in future applications.

Physiologically, the differences in voice features between menstrual phases may be attributed to differences in hormone concentrations. In the follicular phase, estrogen levels rise, whereas the luteal phase is characterized by increased progesterone levels. The larynx itself is a sexual organ and can be influenced by reproductive hormones, as estrogen stimulates the mucosal cells. Mucus on the vocal cords has been shown to be similar in consistency and makeup as cervical mucus throughout the menstrual cycle, displaying increased thin, wet mucus in the follicular phase and thick, dry mucus in the luteal phase [27]. A thin, wet mucus layer is necessary for proper vocal cord vibration and range of pitch [28], and decreased vocal range has been associated with thicker, dry laryngeal mucus [27,29]. Our results align well with these depictions, as we observed a decrease in the F0SD and range (observed through an increase in the minimum F0) in the luteal phase. An alternative explanation for vocal changes may reside in more nuanced apps. Fatigue and stress may be exacerbated in the luteal phase [30], and increased fatigue has been associated with increased F0SD [31]. Regardless, changes in mental and hormonal states associated with the changing menstrual phases provide evidence for the observed vocal changes.

We found that individually scaled F0 voice features had distinct differences between the follicular and luteal phases. Similar to what was observed in previous studies, F0SD decreased and F0MIN increased in the luteal phase [14,15]. Meanwhile, the association between the F0AV and the menstrual phase was insignificant. F0AV throughout the menstrual cycle has traditionally reported mixed results, in which some authors found that F0 increased in periods of high fertility [8,32], decreased in high fertility [15,33,34], or did not change [6,9,14,35-39]. This difference may be attributed to different study methodologies, such as recording a vowel instead of a fixed phrase or only recording 1 voice sample per menstrual phase.

Changes in the voice surrounding the fertile period have been hypothesized to be the result of evolutionary processes. More attractive female participants have an increased likelihood of finding a suitable mate, and mating during the fertile window has the highest likelihood of conception [1]. Research has shown that variations in female voice characteristics throughout the menstrual cycle can influence perceived attractiveness. In the high fertility days prior to ovulation, female voices have typically been rated “more attractive” by heterosexual male participants [15,16]. It has been shown that 44%‐55% of the variance in the desirability scores for female participants was explained by their minimum F0 in speech. This may indicate that female voices with lower pitch minima, opposed to a change in the mean pitch, are more desirable [40]. Our findings align nicely with this result, as the 5th percentile of the pitch was an average of 8.8% lower in the follicular phase (a phase corresponding to higher fertility) compared to the luteal phase for all our participants. Furthermore, increased pitch variability, similar to what was seen in the F0SD in the follicular phase, has been associated with increased social attractiveness of the speaker [41,42].

One of the primary distinctions of our work is the employment of a changepoint detection algorithm to identify the day of the switch in vocal features within the menstrual cycle. Although the average predicted changepoints fell within 2 days of ovulation in all participants, there was a large variation in the values that would not permit accurate ovulation prediction. However, when assessing the changepoints in the broader sense of fertility identification, we note that changepoints are identified within the fertile window in a high proportion of participants. The fertile window is associated with an increased likelihood of conception, making it a meaningful target for detection, even if exact ovulation timing is not achieved [1]. In particular, we observed that changepoints are detected within the fertile window in up to 81% (n=13) of participants for F0SD and F0MIN. However, this result was not observed for F0AV and F0MAX.

Offline changepoint detection is a useful method for fertility monitoring, even without the real-time benefits of online detection (in which changepoints can be detected as the data are collected). Current fertility monitoring methods can also require daily use or cycle-long data collection to accurately pinpoint the timing of ovulation. For example, BBT is used as an ovulation confirmation, in which the BBT must be measured every day for at least 1 full menstrual cycle to identify ovulation [1]. After further refinement, offline detection used in a consumer product would have similar confirmatory utility.

Unlike previous studies that had limited voice data collection methodologies, our approach reflects real-world situations by using at-home smartphone recordings, allowing for a more comprehensive assessment of voice parameters throughout the menstrual cycle. This opens up avenues for exploring the feasibility of using voice parameters as indicators of high fertility and ovulation. Moreover, the simplicity and noninvasiveness of smartphone-based recording make it suitable for widespread use in both clinical settings and personal health monitoring. With just a voice and a smartphone app, users do not require any additional devices, highlighting the convenience and accessibility of this method.

### Limitations

Our study’s insights into vocal pitch changes across the menstrual cycle are limited by a small sample size (n=16 participants), which may not capture the full spectrum of variability across a wider population. Additionally, analyzing recordings from only 1 menstrual cycle per participant neglects potential intraindividual variability across different cycles. Finally, our examination of a limited set of pitch features restricts our understanding of the nuanced relationship between vocal characteristics and menstrual phases, and there may be more information located in other pitch-related features. With further refinement through larger sample sizes, multicycle analyses, and a broader examination of vocal features, we could gain better insights into how vocal pitch correlates with menstrual cycle changes. Importantly, using larger, multicycle datasets could enable the use of online changepoint detection algorithms, which have the advantage of identifying phase transitions in real-time without requiring access to the entire dataset beforehand, or could be used to train a machine learning model for enhanced performance. With these advancements, this method could contribute to the early detection of fertility issues, personalized fertility tracking, and natural family planning.

### Conclusions

Our study contributes to the evolving understanding of how menstrual cycle phases impact vocal pitch, underscoring the potential of voice as a noninvasive marker for fertility and ovulation. Using methods that use real-world data captured through smartphone recordings, our study analyzed these data with changepoint detection algorithms to identify transitions between menstrual phases. This approach not only confirms the relationship between vocal pitch variations and the menstrual cycle phase but also demonstrates the feasibility of applying such findings in practical, real-world settings. Our work could have implications for clinical and personal health monitoring, offering potential improvements in fertility tracking and natural family planning methods. Future efforts to integrate more sophisticated machine learning techniques and real-time analysis could further refine the accuracy and applicability of vocal pitch as a tool for women’s health management.

## Abbreviations

BBT: basal body temperature
F0: fundamental frequency
F0AV: mean fundamental frequency
F0MAX: 95th percentile of the fundamental frequency
F0MIN: 5th percentile of the fundamental frequency
F0SD: fundamental frequency SD
LH: luteinizing hormone

## References

[1] Su HW, Yi YC, Wei TY, Chang TC, Cheng CM (2017). Detection of ovulation, a review of currently available methods. Bioeng Transl Med.

[2] Johnson S, Marriott L, Zinaman M (2018). Can apps and calendar methods predict ovulation with accuracy?. Curr Med Res Opin.

[3] Haselton MG, Gildersleeve K (2016). Human ovulation cues. Curr Opin Psychol.

[4] Haselton MG, Gildersleeve K (2011). Can men detect ovulation?. Curr Dir Psychol Sci.

[5] Zamponi V, Mazzilli R, Mazzilli F, Fantini M (2021). Effect of sex hormones on human voice physiology: from childhood to senescence. Hormones (Athens).

[6] Amir O, Biron-Shental T (2004). The impact of hormonal fluctuations on female vocal folds. Curr Opin Otolaryngol Head Neck Surg.

[7] Silverman EM, Zimmer CH (1978). Effect of the menstrual cycle on voice quality. Arch Otolaryngol Head Neck Surg.

[8] Bryant GA, Haselton MG (2009). Vocal cues of ovulation in human females. Biol Lett.

[9] Çelik Ö, Çelik A, Ateşpare A (2013). Voice and speech changes in various phases of menstrual cycle. J Voice.

[10] Shoup-Knox ML, Ostrander GM, Reimann GE, Pipitone RN (2019). Fertility-dependent acoustic variation in women’s voices previously shown to affect listener physiology and perception. Evol Psychol.

[11] Pavela Banai I, Burriss RP, Šimić N (2022). Voice changes across the menstrual cycle in response to masculinized and feminized man and woman. Adapt Human Behav Physiol.

[12] Narasimhan SV, Pooja M (2022). Vocal changes in different phases of menstrual cycle: an evidence from the acoustic, cepstral, and spectral analysis. J Indian Speech Lang Hear Assoc.

[13] Santos SMS (2018). Is there any correlation between voice and human ovulation? [Doctoral Dissertation].

[14] Pavela Banai I (2017). Voice in different phases of menstrual cycle among naturally cycling women and users of hormonal contraceptives. PLoS One.

[15] Fischer J, Semple S, Fickenscher G (2011). Do women’s voices provide cues of the likelihood of ovulation? The importance of sampling regime. PLoS One.

[16] Pipitone RN, Gallup GG (2008). Women’s voice attractiveness varies across the menstrual cycle. Evol Hum Behav.

[17] McFee B, McVicar M, Faronbi D librosa. Zenodo.

[18] Yang P, Dumont G, Ansermino JM (2006). Adaptive change detection in heart rate trend monitoring in anesthetized children. IEEE Trans Biomed Eng.

[19] Malladi R, Kalamangalam GP, Aazhang B Online bayesian change point detection algorithms for segmentation of epileptic activity.

[20] Staudacher M, Telser S, Amann A, Hinterhuber H, Ritsch-Marte M (2005). A new method for change-point detection developed for on-line analysis of the heart beat variability during sleep. Physica A Stat Mech Appl.

[21] Bosc M, Heitz F, Armspach JP, Namer I, Gounot D, Rumbach L (2003). Automatic change detection in multimodal serial MRI: application to multiple sclerosis lesion evolution. Neuroimage.

[22] Kass-Hout TA, Xu Z, McMurray P (2012). Application of change point analysis to daily influenza-like illness emergency department visits. J Am Med Inform Assoc.

[23] Aminikhanghahi S, Cook DJ (2017). A survey of methods for time series change point detection. Knowl Inf Syst.

[24] Truong C, Oudre L, Vayatis N (2020). Selective review of offline change point detection methods. Signal Processing.

[25] McCracken JA, Custer EE, Lamsa JC (1999). Luteolysis: a neuroendocrine-mediated event. Physiol Rev.

[26] Seabold S, Perktold J Statsmodels: econometric and statistical modeling with python.

[27] Abitbol J, Abitbol P, Abitbol B (1999). Sex hormones and the female voice. J Voice.

[28] Levendoski EE, Leydon C, Thibeault SL (2014). Vocal fold epithelial barrier in health and injury: a research review. J Speech Lang Hear Res.

[29] Bonilha HS, White L, Kuckhahn K, Gerlach TT, Deliyski DD (2012). Vocal fold mucus aggregation in persons with voice disorders. J Commun Disord.

[30] Li SH, Lloyd AR, Graham BM (2020). Physical and mental fatigue across the menstrual cycle in women with and without generalised anxiety disorder. Horm Behav.

[31] Vogel AP, Fletcher J, Maruff P (2010). Acoustic analysis of the effects of sustained wakefulness on speech. J Acoust Soc Am.

[32] Tatar EC, Sahin M, Demiral D (2016). Normative values of voice analysis parameters with respect to menstrual cycle in healthy adult Turkish women. J Voice.

[33] Amir O, Kishon-Rabin L, Muchnik C (2002). The effect of oral contraceptives on voice: preliminary observations. J Voice.

[34] Karthikeyan S, Locke JL (2015). Men’s evaluation of women’s speech in a simulated dating context: effects of female fertility on vocal pitch and attractiveness. Evol Behav Sci.

[35] Barnes L, Latman N (2011). Acoustic measure of hormone affect on female voice during menstruation. Int J Humanit Soc Sci.

[36] Chae SW, Choi G, Kang HJ, Choi JO, Jin SM (2001). Clinical analysis of voice change as a parameter of premenstrual syndrome. J Voice.

[37] Figueiredo LCD, Gonçalves MIR, Pontes A, Pontes P (2004). Vocal behavior during menstrual cycle: perceptual-auditory, acoustic and self-perception analysis. Rev Bras Otorrinolaringol.

[38] Meurer EM, Garcez V, von Eye Corleta H, Capp E (2009). Menstrual cycle influences on voice and speech in adolescent females. J Voice.

[39] Raj A, Gupta B, Chowdhury A, Chadha S (2010). A study of voice changes in various phases of menstrual cycle and in postmenopausal women. J Voice.

[40] Pisanski K, Oleszkiewicz A, Plachetka J, Gmiterek M, Reby D (2018). Voice pitch modulation in human mate choice. Proc Biol Sci.

[41] Ray GB, Ray EB, Zahn CJ (1991). Speech behavior and social evaluation: an examination of medical messages. Commun Q.

[42] Zuckerman M, Miyake K (1993). The attractive voice: what makes it so?. J Nonverbal Behav.

